# Canopy Architecture and Sun Exposure Influence Berry Cluster–Water Relations in the Grapevine Variety Muscat of Alexandria

**DOI:** 10.3390/plants13111500

**Published:** 2024-05-29

**Authors:** Olfa Zarrouk, Clara Pinto, Maria Victoria Alarcón, Alicia Flores-Roco, Leonardo Santos, Teresa S. David, Sara Amancio, Carlos M. Lopes, Luisa C. Carvalho

**Affiliations:** 1 LEAF—Linking Landscape, Environment, Agriculture and Food Research Center, Associate Laboratory TERRA, School of Agriculture, University of Lisbon, Tapada da Ajuda, 1349-017 Lisboa, Portugalsamport@isa.ulisboa.pt (S.A.); carlosmlopes@isa.ulisboa.pt (C.M.L.); 2 IRTA—Institute of Agrifood Research and Technology, Torre Marimon, 08140 Barcelona, Spain; 3 INIAV—Instituto Nacional de Investigação Agrária e Veterinária, I.P. Avenida da República, Quinta do Marquês, 2780-159 Oeiras, Portugal; clara.pinto@iniav.pt (C.P.); teresa.david@iniav.pt (T.S.D.); 4 CEF—Forest Research Centre, Associate Laboratory TERRA, School of Agriculture, University of Lisbon, Tapada da Ajuda, 1349-017 Lisboa, Portugal; 5 Area of Agronomy of Woody and Horticultural Crops, Centro de Investigaciones Científicas y Tecnológicas de Extremadura (CICYTEX), 06187 Badajoz, Spain; maria.alarcon@juntaex.es (M.V.A.); alicia.flores.roco@usc.es (A.F.-R.)

**Keywords:** aquaporins, histology, hydraulic conductivity, phenology, stress-related genes, *Vitis vinifera*

## Abstract

Climate-change-related increases in the frequency and intensity of heatwaves affect viticulture, leading to losses in yield and grape quality. We assessed whether canopy-architecture manipulation mitigates the effects of summer stress in a Mediterranean vineyard. The *Vitis vinifera* L variety Muscat of Alexandria plants were monitored during 2019–2020. Two canopy shoot-positioning treatments were applied: vertical shoot positioning (VSP) and modulated shoot positioning (MSP). In MSP, the west-side upper foliage was released to promote partial shoot leaning, shading the clusters. Clusters were sampled at pea size (PS), veraison (VER), and full maturation (FM). Measurements included rachis anatomy and hydraulic conductance (Kh) and aquaporins (AQP) and stress-related genes expression in cluster tissues. The results show significant seasonal and interannual differences in Kh and vascular anatomy. At VER, the Kh of the rachis and rachis+pedicel and the xylem diameter decreased but were unaffected by treatments. The phloem–xylem ratio was either increased (2019) or reduced (2020) in MSP compared to VSP. Most AQPs were down-regulated at FM in pedicels and up-regulated at VER in pulp. A potential maturation shift in MSP was observed and confirmed by the up-regulation of several stress-related genes in all tissues. The study pinpoints the role of canopy architecture in berry–water relations and stress response during ripening.

## 1. Introduction

Reports by the IPCC [1] indicate that the ongoing increase in temperature and decrease in precipitation in Southern Europe are a result of climate change. Projections foresee a marked increase in the frequency and intensity of heatwaves, meteorological droughts, and heavy precipitation events in the region [2,3]. Specifically, diurnal and nocturnal temperatures during the grape growing season will increase, as well as maximum temperatures during the ripening period [4]. These changes, together with the phenomenon of global brightening [2], will increase the incidence of sunburn damage in grape berries, with disastrous economic consequences.

Winemaking is considered one of the most historically relevant socio-economic activities in Portugal, with grapevines representing approximately 14% of the total planted area of the overall agriculture sector and 6% of the total production [5] and wine accounting for nearly 2% of the total national exports. In recent decades, the Portuguese wine industry has been severely affected by climate change, a trend expected to remain unchanged in the near future due to the high sensitivity of grapes to small changes in climatic conditions [6].

Although, in the past, sunburn was not a frequent event in European viticultural regions, historical records show an increased frequency of seasons with significant sunburn damage. In Portugal, this phenomenon has been attributed mainly to a higher frequency and intensity of heat waves [7]. Berry sunburn is a recurring disorder that can reduce berry quality and cause severe yield loss [8]. Canopy management and manipulation of training systems could be used as strategies to protect grape berries from sunburn. Although traditional training management in Southern Europe was designed to provide a certain degree of protection to grapes (e.g., gobelet and pergola), in recent years, vertical shoot-positioned (VSP) canopy systems have become widespread in vineyards in Portugal, mainly because it is a training system very well adapted to mechanization. Historically, VSP training systems were designed in traditional vineyards in central Europe to increase fruit exposure. Consequently, when used in hot and dry terroirs, VSP risks overexposing clusters [9].

Different short-term solutions have been implemented to reduce yield losses due to sunburn in VSP grapevines in the hottest winegrowing regions, including the use of netting, particle-film-forming products, anti-transpirants, and hydrocooling. However, the results of the effectiveness of these solutions in viticulture are still inconclusive (reviewed in [10]). These solutions also increase vineyard management costs and potentially increase the vineyard’s carbon and/or water footprint. Minimal pruning systems are employed in the hottest winegrowing regions and provide sufficient shelter to protect grapes from sunburn, although several reports warn that this type of management is prone to producing smaller-sized clusters and modifying oenological characteristics [11,12]. Alternative manipulation of north–south oriented VSPs by modulating the shoot position on the west side of the canopy could provide adequate protection to the exposed clusters and reduce sunburn incidence, as it keeps clusters under a diffuse light regime and decreases direct radiation. This management could be easily applied by grapevine growers and could be of high significance in sunburn-susceptible cultivars, such as Riesling or Muscat of Alexandria [13]. However, the role of irradiance in the control of shoot and leaf hydraulic conductivity [14] suggests that increasing berry shading could influence the water relations between the parent plant and the berry clusters. Furthermore, the ability to maintain berry turgor is related to resistance to shriveling [10].

Grape growth is mainly the result of water accumulation, and therefore, its maintenance requires the concerted action of long-distance water and solute transport through vascular tissues (which connect the fruit to the parent plant) and short-distance water and solute uptake by individual cells [15]. The role of vascular tissues in grape berries has been a subject of research for several decades, with the aim of deciphering the basis of cluster and berry–water relations [16]. It has been demonstrated that water enters the fruit predominantly via the xylem before veraison, after which xylem transport declines gradually, and the phloem provides most of the water during the ripening stages of the fruit [17]. Nonetheless, several biological questions regarding the mechanisms underlying berry weight loss and other ripening disorders remain unanswered. Currently, the cessation of xylem flow into berries at veraison is conventionally accepted. However, data from dye-tracing studies in pedicels have proven that the xylem remains physically intact [18,19], enabling water backflow from the fruit to the parent plant when the water inflow exceeds the elastic modulus of fruits [20]. In addition, several studies on fleshy fruits have shown the detrimental effects of late-season rain or excessive irrigation on the late stages of fruit ripening [21,22,23]. More recently, McElrone et al. [24] reported that grapevine exhibits functional hydraulic sectoring and confirmed that the xylem remains conductive into the berry through ripening, but only the clusters attached to hydraulic sectored shoots had these direct connections. These data opened the debate on the extent to which viticultural management and environmental events could disrupt water relations between plants and fruits. However, post-vascular transmembrane water transport mediated by aquaporins (AQP) should also be considered, since differential expression of AQP genes occurs during fruit development [25,26]. Aquaporins are related to changes in xylem hydraulic resistance during berry maturation or to the accumulation of sugars at post-veraison stages (Sabir et al. [27]). Particularly, the modulation of gene expression in different classes of AQPs has been associated with the hydraulic buffering of grape berries from the parent plant at veraison [19,26]. Furthermore, AQPs have been related to the regulation of water homeostasis in light-adapted leaves in bur oak [28]. This suggests that differential AQP expression in berries due to canopy modulation and light interception could have a critical impact on berries/cluster turgor and hydraulic properties, ripening processes, and quality. However, to the best of our knowledge, the influence of canopy-architecture manipulation in cluster water relations and in the mitigation of the characteristic abiotic stress conditions of the Mediterranean summer season are yet to be thoroughly addressed.

Therefore, the present study aims to assess the responses of sunburn-sensitive Muscat of Alexandria grapevines to short-term adaptation strategies, such as different shoot-training systems, e.g., modulated shoot positioning (MSP), that are expected to balance grape-berry growth regulation and sunburn protection. By combining physiological (hydraulic conductance), anatomical (rachis vascular tissues), and molecular (AQP and stress-related gene expression) approaches, we aim to decipher how VSP and MSP impact water inflow in grape-berry clusters and contribute to reducing sunburn effects in vineyards with varieties sensitive to sunburn.

## 2. Results

### 2.1. Histochemical Analysis of Peduncles

**Figure 1:**
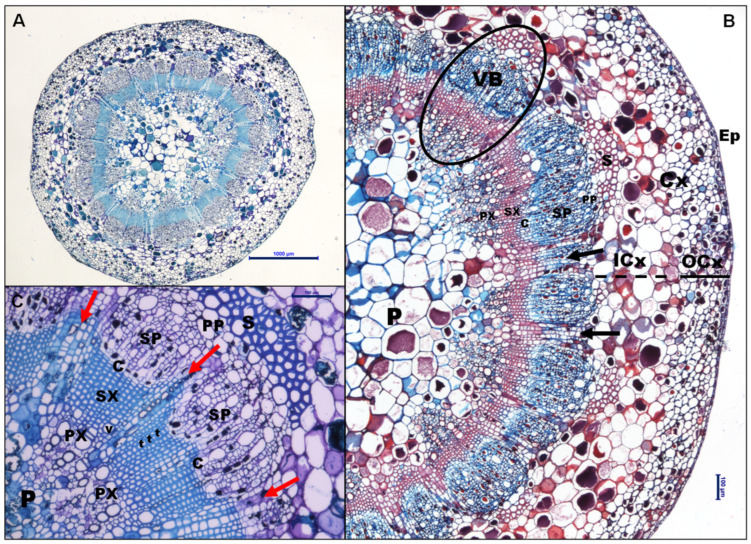
Anatomy of peduncle. (**A**) Complete cross section of FM in VSP from 2019, stained with Toluidine Blue. Scale bar: 1000 μm. (**B**) Cross section of PS in MSP from 2020 stained with Safranin and Astra Blue. Both lignified secondary xylem cells and sclerenchyma cells turn reddish with safranin. Scale bar: 100 μm. (**C**) Details of a vascular bundle of VER in MSP from 2020 stained with Toluidine Blue. Scale bar: 100 μm. Arrows indicate radio-medullary parenchyma rays. Abbreviations: C: cambium; Cx: cortex; OCx and ICx indicate the outer and inner layers of cortex parenchyma. Ep: epidermis; P: pith; PP: primary phloem; PX: primary xylem; S: sclerenchyma; SP: secondary phloem; SX: secondary xylem; t: tracheid; v: vessel; VB: vascular bundle.

Transverse sections of the peduncle showed identical tissue organization in VSP and MSP at the three phenological stages (Figure 1A). The external organization of the sections is composed of a monostratified layer of small isodiametric epidermal cells (Ep) covered by a thin cuticle (Figure 1B). The cortex parenchyma is divided into two layers: The outer layer (OCx) of small well-organized cells below the epidermis, and the inner layer (ICx) of larger cells surrounding the vascular cylinder (Figure 1B). The vascular bundles are collaterals, with an average of 22–24 vascular bundles, in which the xylem and phloem are arranged in a specific radial pattern (Figure 1C). In particular, the peduncle is maintained in an intermediate state between primary and secondary growth. Transverse sections also showed that the epidermis is not continuous, contains stomata, and has the formation of small lenticels (Figure S1).

**Figure 2:**
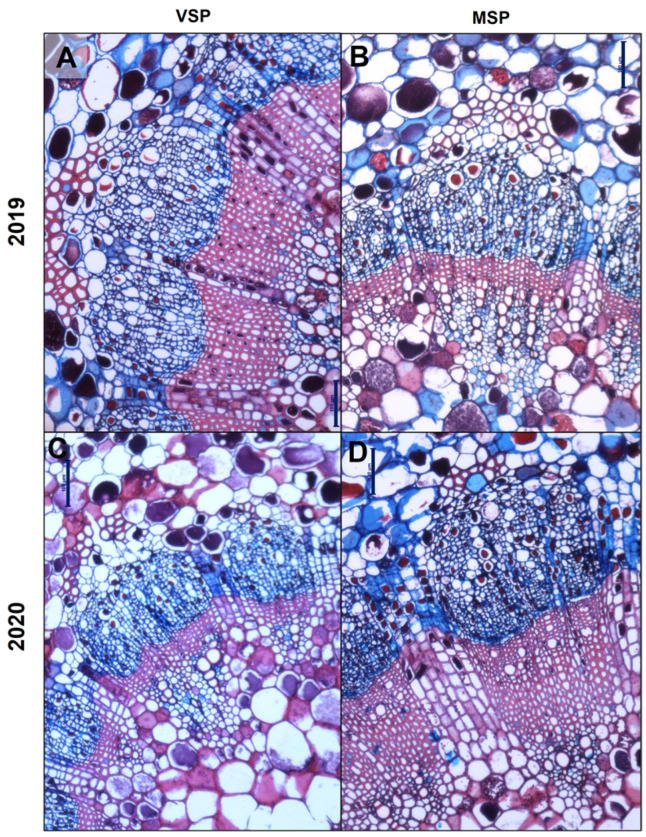
Details of vascular tissue areas at FM stained with Safranin and Astra Blue. In 2019, a significant decrease in xylem and phloem areas in MSP (**B**) when compared to VSP (**A**). In 2020, the xylem area is increased in MSP (**C**) versus VSP (**D**). Scale bars: 100 μm.

The comparative study of the effect of shoot position on peduncle cell parameters (MSP vs. VSP) in the 2019 and 2020 seasons is shown in Figure 2 and, with more detail, in Table S1. In both seasons, the peduncle sections’ total areas, together with the areas occupied by the different tissues (pith, xylem, phloem cortex, and epidermis), were measured, and their relative proportion was calculated. The results showed that the area occupied by the different tissues (cortex, vascular cylinder, xylem, phloem, and pith) decreased at VER compared to PS, which may indicate a halt to growth.

**Figure 3:**
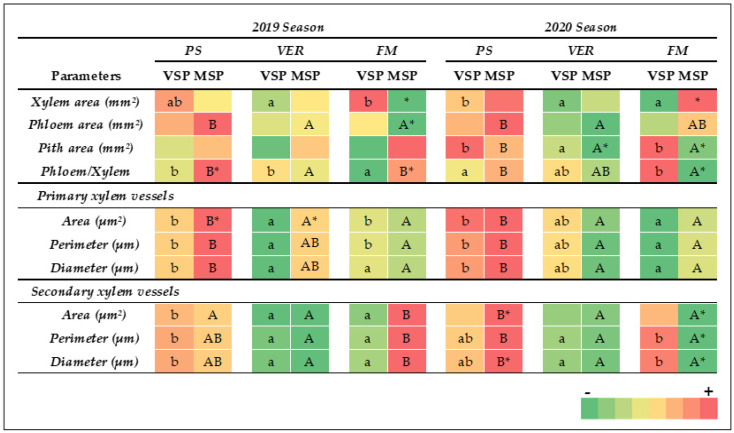
Morpho-anatomical parameters and quantitative characteristics of vascular tissue of Muscat of Alexandria peduncle at three developmental stages during the 2019 and 2020 seasons under different shoot-positioning treatments (VSP and MSP) at three different phenological stages (pea size, PS; veraison, VER; and full maturation, FM). For each parameter, different letters indicate significant differences between sampling times for VSP (lower-case) or MSP (upper-case), while * indicates significant differences between treatments at the same sampling time and are indicated in the MSP square (ANOVA and Tukey’s HSD. *p* < 0.05).

Overall, the treatment MSP did not induce a significant change in the sectional area, the cortex, and the vascular cylinder in all phenological stages. However, a significant effect of MSP on the total vascular tissue areas (phloem and xylem) was observed. The xylem area did not suffer changes during the three phenological stages in both treatments in both seasons, except for VSP in 2019, where the lowest xylem area values were observed at VER. The phloem area showed conserved values during berry maturation in VSP both in 2019 and 2020. However, in MSP, the phloem area was reduced from PS onward in both 2019 and 2020 (Figure 3). At FM, MSP showed a significant decrease in xylem (1.61 ± 0.07 vs. 1.11 ± 0.09) and phloem (1.52 ± 0.06 vs. 1.32 ± 0.07) areas when compared to VSP in 2019 (Figure 2A,B). However, in 2020, MSP showed a higher xylem area at FM (1.18 ± 0.10 vs. 1.81 ± 0.10) (Figure 2C,D). These contrasting results between seasons impacted the phloem–xylem ratio, which was significantly higher at FM in MSP in 2019 but lower in 2020 when compared to VSP (Figure 3).

In general, the primary xylem vessel area, perimeter, and diameter significantly decreased at VER and at FM compared to PS in both treatments and years. When compared to VSP, MSP significantly increased the primary xylem area at VER in 2019 (Figure 2). In contrast, no significant difference between VSP and MSP was observed on the primary xylem parameters in 2020 (Figure 2). At VER, all measured secondary xylem parameters decreased when compared to PS in both treatments and years. At FM, all parameters (area, perimeter, and diameter) of secondary xylem vessels increased under MSP in 2019 and decreased significantly in 2020 (Figure 2).

### 2.2. Hydraulic Conductance

**Figure 4:**
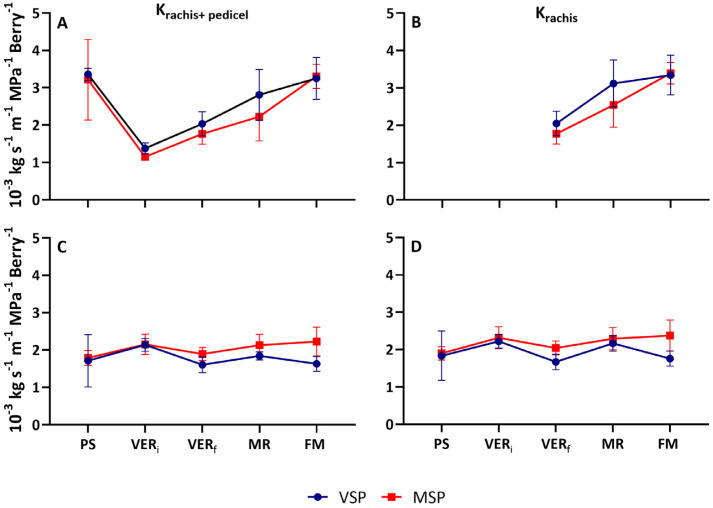
Specific hydraulic conductivity Kh (Kg s^−1^ MPa^−1^ m^−1^ Berry^−1^) in cluster (rachis+pedicel) and in rachis (normalized to cluster length and berry number) at five phenological stages (pea size (PS), beginning of veraison (VER_i_), end of veraison (VER_f_), mid-ripening (MR), and full maturation (FM)) of the Muscat of Alexandria variety conducted in VSP and MSP in 2019 (**A**,**B**) and 2020 (**C**,**D**) seasons. Data are means ± SE (*n* = 5).

Different cluster hydraulic dynamics during berry maturation were observed between seasons (Figure 4). However, no differences in pattern were observed in the hydraulic conductance of rachis and pedicel (K_rachis+pedicel_) and the hydraulic conductance of rachis (K_rachis_) between the two treatments, VSP and MSP.

In the 2019 season, K_rachis+pedicel_ was high at PS and decreased significantly thereafter (≈60% in VSP and ≈65% in MSP), showing minimal values at VER_i_. After veraison, K_rachis+pedicel_ increased in both treatments, showing maximal values at FM (Figure 4A). After veraison, K_rachis_ increased both in VSP and MSP (Figure 4B). Although no significant differences were observed between treatments in all assessed stages, MSP showed a slow increase in hydraulic, compared with VSP.

In the 2020 season, both K_rachis+pedicel_ and K_rachis_ showed a steady-state behavior during berry ripening (Figure 4C,D). A slight decrease (≈25% in VSP and ≈10% in MSP) at VER and a subsequent minimal increase at FM of both parameters were observed.

**Figure 5:**
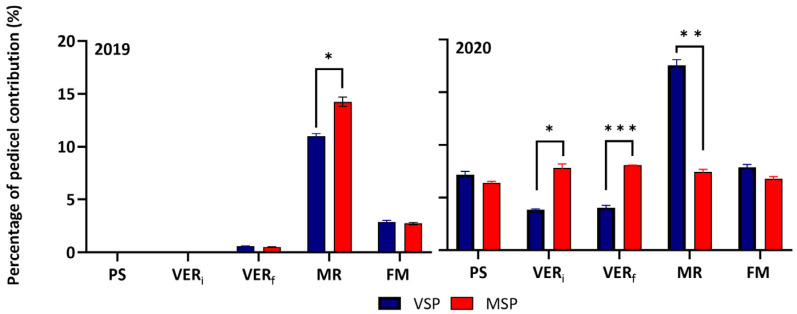
Pedicel conductivity contribution to the total cluster (rachis+pedicel) hydraulic conductivity (%) at five phenological stages (pea size (PS), beginning of veraison (VER_i_), end of veraison (VER_f_), mid-ripening (MR), full maturation (FM)) of the Muscat of Alexandria variety trained in VSP and MSP during 2019 and 2020 seasons. Data are means ± SE (*n* = 4). Comparisons between treatments at the same sampling time were performed by Student’s *t*-test (*: *p* < 0.05; **: *p* < 0.01; ***: *p* < 0.001).

In both seasons, pedicels exerted a strong control of water flow in all stages. The pedicel hydraulic conductivity, derived from the difference between K_rachis_ and K_pedicel+rachis_, was lowest around VER (VER_i_ and VER_f_) and highest at MR in both seasons. Interestingly in 2020, the contribution of pedicels in the total conductivity of the cluster showed a steady-state behavior in MSP during the berry development stages, while it was modulated by phenology in VSP (Figure 5).

### 2.3. Gene Expression

As mentioned above, the largest trend changes in hydraulic conductivity during berry maturation were observed in 2019 compared to 2020. Consequently, to understand the role of aquaporins (AQP) in water movement and hydraulics within the reproductive structures and tissues, AQP expression was performed on the 2019 samples. Also, the pattern of expression of some stress-related genes was monitored solely in 2019.

#### 2.3.1. Aquaporins

In general, AQPs showed differential expression among tissues and developmental stages, with different regulation responses to the treatment; the extent of changes in expression was more significant in the pedicel and pulp compared to the skin.

**Figure 6:**
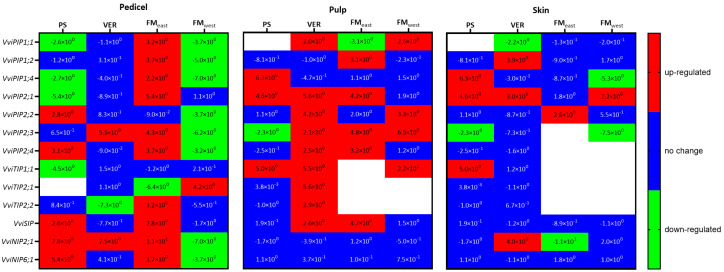
Expression of genes coding for aquaporins of the PIP (plasma membrane intrinsic proteins), TIP (tonoplast intrinsic proteins), NIP (nodulin26-like intrinsic proteins), and SIP (small basic intrinsic proteins) subfamilies (log_2_(fold change)) during the berry maturation stages (pea size (PS), veraison (VER), and full maturation both at east (FM_east_) and west side (FM_west_)) in pedicel, pulp, and skin of the Muscat of Alexandria variety. Relative values for the treatmentsMSP are expressed in comparison to VSP. White boxes correspond to not-detected gene expression.

As shown in the heat map in Figure 6, the highest expression levels of most AQP genes were attained during the first stages of berry ripening (PS and VER) and decreased thereafter in FM.

In the pedicel, the genes coding PIP1 and PIP2 isoforms showed a contrasting response to the treatment at PS. PIP1 isoforms were, in general, down-regulated (except for *VviPIP1;2*), while PIP2 were up-regulated (except for *VviPIP2;3*, which was not affected by the treatment, and *VviPIP2;1*, which was significantly down-regulated in MSP). Concerning TIPs, *VviTIP1;1* was significantly down-regulated at PS, while no expression of *VviTIP2;1* was observed in both treatments. On the other hand, the SIP and NIP families were up-regulated in MSP. At VER, no significant expression changes were observed between treatments, except for *VviPIP2;3* and *VviNIP2;1*, which were up-regulated in MSP, and *VviTIP2;2*, which was highly down-regulated in MSP. At FM, differences between the two sides of the canopy were observed. At FM_west_ (side shaded by the canopy in MSP), all PIPs and NIPs were down-regulated, while *VviTIP2;1* was up-regulated. In contrast, at FM_east_, most AQPs were up-regulated, except for *VviPIP2;2* and *VviTIP1;1,* which showed no change, and *VviTIP2;1*, which was down-regulated.

At PS *VviPIP1;4*, *VviPIP2;1* and *VviTIP1;1* were up-regulated in MSP, while *VviPIP2;3* was down-regulated. *VviPIP1;1* was not detected in the berry at this stage.

In the pulp, almost all studied AQPs were up-regulated at VER in MSP, except for *VviPIP1;2*, *VviPIP1;4*, and *VviNIPs*. At FM, *VviPIP1;1*, *VviPIP2;2*, *VviPIP2;3* and *VviTIP1;1* maintained the up-regulation at the west side. At the east side, *VviPIP1;2*, *VviPIP2;1*, *VviPIP2;3*, *VviPIP2;4*, and *VviSIP* were up-regulated, while *VviPIP1;1* was down-regulated. It is worth noting that *VviTIP1;1* expression was not detected in VSP at FM and in MSP on the east side, while it was highly expressed in MSP on the west side. In contrast, *VviTIP2;1* expression was repressed in MSP on both sides. *VviTIP2;2* was no longer expressed in the pulp at FM in both treatments.

Compared with the other organs, in the skin, few AQPs showed differential expression response between treatments. At VER, *VviPIP1;1* was down-regulated in MSP, while *VviPIP1;2*, *VviPIP2;1*, and *VviNIP2;1* were up-regulated. At FM, few changes occurred on the east side, with the up-regulation of *VviPIP2;2* and the down-regulation of *VviNIP2;1* in MSP. In FM_west_ *VviPIP1;2* was up-regulated, while *VviPIP1;4* and *VviPIP2;3* were significantly down-regulated. No expression was recorded in the skin for *VviPIP2;4* and *VviTIPs*. No *VviTIPs* were detected in FM on both sides of the canopy.

#### 2.3.2. Stress-Related Genes

**Figure 7:**
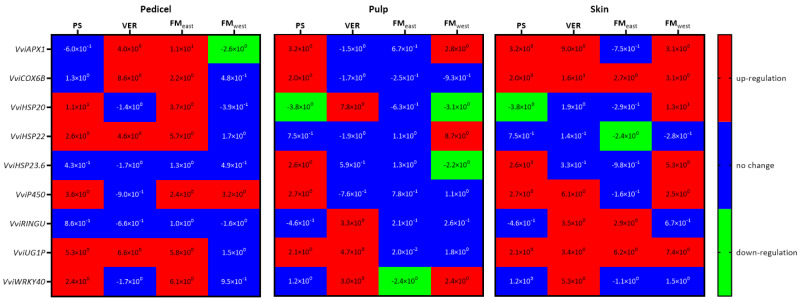
Stress-related genes *VviAPX1*, *VviCOX6B*, *VviHSP20*; *VviHSP22*, *VviHSP23.6*, *VviP450*, *VviRINGU*, *VviUG1P*, *VviWRKY40* gene expression (log_2_(fold change)), during the grape maturation stages (pea size (PS), veraison (VER), and full maturation both at east (FM_east_) and west side (FM_west_)) in pedicel, pulp, and skin of Muscat of Alexandria variety. Relative values for the treatment MSP are expressed in comparison to VSP.

As well as AQP, stress-related genes also showed different patterns of expression over time and in each tissue (Figure 7). In pedicels, the most striking pattern was the significant up-regulation of most genes in FM_east_ in MSP when compared with VSP. In fact, the only exceptions were *VviRingU* and *VviHSP23.6*, which suffered no significant changes between treatments. In PS, *VviHSP20*, *VviHSP22*, *VviP450*, *VviUGIP*, and *VviWRKY40* were significantly up-regulated in MSP. In VER, *VviAPX1*, *VviCOX6B*, *VviHSP22*, and *VviUGIP*, were significantly up-regulated in MSP. In those two developmental stages, no genes were significantly down-regulated. Only on FM_west_ was a gene significantly down-regulated in pedicels, *APX1*, while *P450* was up-regulated.

In the pulp, three significantly high levels of up-regulation of sHSPs were observed, *VviHSP23.6* in PS, *VviHSP20* in VER, and *VviHSP22* on FM_west_. In the pulp of FM_east_, contrary to the pattern of the pedicels, no genes were up-regulated, and the only significant change was the down-regulation of *VviWRKY40*. In VER, *VviHSP20*, *VviRingU*, *VviUGIP*, and *VviWRKY40* were up-regulated. Meanwhile, in FM_west_, the three up-regulated genes were *VviAPX1*, *VviHSP22*, and *VviWRKY40*, while *VviHSP20* and *VviHSP23.6* were down-regulated. On PS, *VviAPX1*, *VviCOX6B*, *VviHSP23.6*, *VviP450,* and *VviUGIP* were significantly up-regulated, and *VviHSP20* was down-regulated.

In the skins, only two genes were down-regulated, and they are both *sHPSs*, *VviHSP20* in PS and *VviHSP22* in FM east. *VviCOX6B* and *VviUGIP* were up-regulated in all phenological stages, while *VviWRKY40* was only up-regulated in VER. *VviAPX1* and *VviP450* were up-regulated in all phenological stages but FM_east_. Meanwhile, *HSP23.6* was up-regulated in PS and FM west, and *VviHSP20* was also up-regulated in FM_west_. In contrast, *VviRingU* was up-regulated in VER and FM_east_.

## 3. Discussion

Fruit hydraulics are receiving increasing attention because of the importance of water transport for fruit growth and quality [29]. Berry water transport depends on the pathway resistance between several structures to which the berry is connected (e.g., peduncle, rachis, and pedicel) and the parent plant, as well as on the driving force for water flow. The resistance is generally attributed to the lumen and inter-conduct resistance, and the resistance of the cell membrane [30], which is regulated by water channels and AQPs [27], [31]. Hydraulic measurements on Muscat of Alexandria clusters showed a dynamic change across phenological stages. Particularly, an increase in resistance around VER was observed, corroborating several previous reports [17,26,32]. This decrease in water flow has been attributed to a shift in the pathway of water transport into the berry from the xylem to the phloem [33], due to the hydraulic buffering of grape berries from the parent plant [19,26]. However, the mechanisms that result in hydraulic buffering have not yet been elucidated. Scharwies and Tyerman [34] showed that cluster hydraulic conductivity could change over the ripening process, depending on the genotype’s iso/anisohydric behavior. A decrease in whole-cluster hydraulic conductivity during berry development has been considered a characteristic of anisohydric genotypes [17]. In our study, Muscat of Alexandria showed contrasting trends between years. While hydraulic conductivity (normalized to rachis length and berry number) decreased during berry ripening during the 2019 season, in 2020 no significant changes were observed. Muscat genotypes have been classified as near-isohydric [35], and this behavior could explain the results obtained in 2020 but fails to explain the plants’ behavior in 2019. Regardless of the controversial classification of grapevine genotypes on the iso/anisohydric groups [16,36]), Vandeleur et al. [37] demonstrated the ability of grapevine genotypes to switch their strategy by shifting from a near-isohydric to a near-anisohydric behavior, depending on the soil water content, thanks to the activity of several AQPs. Another hypothesis could be that there was not a complete correspondence in berry developmental status between both seasons. Indeed, the onset of ripening depends not only on the genetics of the genotype but is also highly influenced by environmental conditions [38]. This means that, in different seasons, the same apparent phenological stage, visually assessed, could, in fact, represent different metabolic stages for berries. Indeed, climatic conditions were different in 2019 and 2020 (Figure S1). In June 2019, at PS, meteorological conditions were favorable for grapevine physiology and berry development, with optimal temperatures (below 30 °C) accompanied by a precipitation event of 16 mm. This may have enhanced leaf stomatal opening and, thus, the increase of water flux within the canopy, explaining the high hydraulic conductance of berry clusters observed at PS. In 2020, however, heavy precipitation occurred in April–May (≈200 mm), coinciding with the berry set, which delayed berry maturation by almost 10 days when compared with 2019. This implied that the berry growth period (e.g., PS, VER, and MR) was delayed to later in the season, occurring in a shorter time span than in 2019 and under more stressful conditions due to the high air temperatures and absence of precipitation during summer. These conditions could have affected the cluster xylem development (e.g., no significant changes in primary and secondary xylem after VER), limiting the water transport capacity, which could explain the relatively stable cluster hydraulic conductance after VER in 2020 compared with 2019, when K_h_ increased in VER_f_. Corroborating this later assumption, anatomical data in 2019 shows a reduction in vessel diameters (primary and secondary xylem) around VER, while in 2020, the vessel diameter shows a constant value during berry development. Knipfer et al. [39] observed a decrease in the K_h_ pedicel despite the increase in the xylem area and associated it with blockage due to vessel elements. Hence, our data suggest that conditions that favor water transport at pea size defined the threshold for later-season water transport in clusters. The increase in hydraulic conductivity, observed in VER_f_ in 2019, was somehow unexpected given the common assumption that berries become dependent on the phloem water supply because xylem inflow declines at the onset of ripening [18,40]. Nonetheless, in tomato, xylem water import still remains the dominant pathway throughout fruit ripening [41]. A recent report on grapevine also points to the functionality of the xylem after VER [20] but with the function of sustaining the backflow of excess phloem-derived water. Scharwies and Tyerman [34], on the other hand, suggested a possible xylem water-import reactivation at the end of ripening when phloem import ceases. In all cases, the lag phase of berry growth, occurring prior to VER, is likely to affect xylem development, with new vessels having a smaller diameter than those produced before and after this stage.

Environmental impacts may be mediated by changes in the water potential and osmotic gradients of the stem–pedicel–fruit continuum. In this sense, low light intensity was shown to decrease the total sap flow and increase the relative contribution of the xylem to the import of fruit water [42,43]. It is, thus, expected that MSP would change water flow in the berry clusters. The absence of significant differences in hydraulic conductance between VSP and MSP in both seasons may result from the timing of the treatment application. As K_h_ is a relatively conservative trait, the fact that MSP was only applied at the PS stage resulted in a short time span to induce significant hydraulic adaptations capable of explaining changes in water flow through the xylem.

According to Pace et al. [44], when fibers increase in abundance, fiber bands become more closely arranged, leading the elements towards a tangential disposition, while the axial parenchyma reduces in abundance and becomes sieve-tubecentric. The presence of this sieve-tubecentric axial parenchyma in the fibrous species may contribute fundamentally to phloem transport by creating the osmotic pressure known to be necessary to maintain turgor pressure for phloem loading and unloading [45,46,47]. On the other hand, different studies reported the involvement of AQPs in changing xylem hydraulic resistance during maturation and/or the accumulation of sugars at VER_f_ (see review by Sabir et al., [27]). Accordingly, AQPs showed differential expressions among organs and developmental stages. Interestingly, MSP treatment modulated the expression of the different AQP genes in all tissues, particularly at the pedicel and pulp levels and with less extent in berry skin. Previous reports showed greater membrane water permeability of PIP2s compared with PIP1s in the yeast system. In grapevine, the ability of water conductance was only demonstrated for *VviPIP2;1*, while the remaining members, *VviPIP2;2*, *VviPIP2;3*, *VviPIP1;1*, and *VviPIP1;4*, did not affect yeast water transport despite their correct localization in the plasma membrane [48]. In addition, reports have shown that the water permeability of grapevine *VviTIPs* is generally higher than in *VviPIPs* [48]. It is worth noting that *VviPIP2;1* and *VviTIP1;1* were down-regulated at PS in the MSP pedicel but highly up-regulated in the berry, indicating the opposite effect of canopy-architecture modulation (MSP) in the cell-to-cell water transport among organs. These later results contrast with reports associating the loss in pedicel osmotic potential over fruit development with a decline in aquaporin activity in the pedicel [39], based on what had been observed for some isoforms in the berry [25,26]. The fact that several AQPs able to transport water were down-regulated at the pedicel at the early stages of berry ripening in MSP, but up-regulated at full maturation (e.g., all AQPs but *VviPIP2;2*, *VviTIP1;1*, and *VviTIP2;1* on the east side and *VviTIP2;2* on the west side) indicate that the treatment induces changes in water relations within the berry cluster during berry maturation in the same plant. The up-regulation of AQPs at FM could be related to high water content in the apoplast driven by the phloem at late ripening to sustain the accumulation of sugars in fruits, as suggested by Keller et al. [40]. This is an indication that MSP stimulates sugar translocation to the berry compared with VSP. This result is sustained by the up-regulation of *VviUG1P* at FM_east_ and FM_west_ in MSP berry skins. The X1 isoform of UTP-glucose-1-phosphate uridyltransferase (UG1P), coded by *VviUG1P* [49], is involved in the synthesis and degradation of sucrose [50]. As such, *VviUG1P* plays an important role in the accumulation of sugars in the berry, selectively channeling the mobilization of sucrose to promote its accumulation in berries [49,51]. Notwithstanding, *VviUG1P* was, in contrast, down-regulated in MSP at VER, suggesting a delay in the accumulation of sugars by the shade effect to a more advanced phenological stage (up-regulation at FM). The results suggest that artificial shading may induce a higher accumulation of sugars after VER, through the activity of *VviUG1P* and AQPs, when compared to exposure to direct sunlight.

In addition, AQP expression may be related to the detoxification process, as part of the oxidative burst around VER [52]. Several grapevine AQPs showed capacity for hydrogen peroxide (H_2_O_2_) transport across membranes [27]. Our data corroborate this hypothesis, since several AQPs related to H_2_O_2_ transport increased their expression in the berry at VER, particularly in pulp. Interestingly, these AQPs were up-regulated in MSP. In addition, the up-regulation of *VviAPX1* in the three tissues at PS and/or VER, known for its activity in scavenging H_2_O_2_ and metabolic processes such as berry maturation [53], suggests that, together with AQPs, it may contribute to a higher activity of elimination and regulation of H_2_O_2_ levels compared to the conditions of direct sunlight. Also, it may prevent excessive accumulation of ROSs during the maturation of the berries and, thus, exert a protective effect against oxidative damage, particularly in the organelles.

Considering that the ripening process in general is associated with large increases in sugar transport and accumulation with changes in cell-wall metabolism [54] and turgor, which are mediated by AQP and their modulation of membrane water permeability, our results indicate an effect of MSP on the ripening process of the berries. The shading provided by MSP may have altered the cluster microclimate at the level of temperature and relative humidity, which may, in turn, have impacted upon the vapor pressure deficit (VPD) with consequences in fruit transpiration rate and thus changes in water demand and flow in the rachis and pedicel. This could explain the down-regulation of most of the AQPs in the pedicel at FM_west_ and not at FM_east_. The up-regulation of *VviCOX6B* only at FM in MSP confirms the microclimate change induced by this treatment. COX6 plays an essential role in the assembly of cytochrome c oxidase (COX), which is involved in electron transport in the mitochondrial respiratory chain [18], and its expression is associated with the absence of light and the presence of sugars [55]. Thus, the up-regulation of *VviCOX6B* in MSP skins and in pedicels may be related to the lower luminosity provided by the artificial shading of the canopy and/or to an increased availability of sugars that may contribute to higher mitochondrial function in the skins and pedicels [56]. This also suggests that the respiration of VSP pedicels is starved, probably due to high temperature and radiation incidence. Pedicels are major pathways for O_2_ diffusion into the grape berry; the decrease, or the halt, of respiration in pedicels reduces O_2_, leading to hypoxia and berry shrivel [57].

Notably, the absence of expression of some AQPs at some phenological stages highlights their specific seasonal function in each organ. In particular, the lack of TIP expression at full maturation both in the pulp (except for *VviTIP1;1*) and the skin could be related to the reduction of vacuolar osmotic pressure in relation to apoplastic pressure, as shown by Keller et al. [40], thereby reducing TIP recruitment for vacuolar homeostasis in these tissues. The high up-regulation of *VviTIP1;1* at FM in MSP suggests the effect of the shading treatment in cell water movement and corroborates the hypothesis of a maturation shift in MSP-treated plants.

Heat-shock proteins (HSPs) are related to the plant’s ability to acquire thermotolerance. HSP expression was shown to be activated and/or increased under high-temperature stress in several organs/tissues [58,59,60], and also under other abiotic stresses [61,62], as well as by fruit developmental processes [63]. The expression pattern of the three studied HSPs differed among berry organs and tissues and was modulated by MSP, indicating their specific role in each phenology and berry compartment. Interestingly, *VviHSP20* and *VviHSP22* were up-regulated at PS in the pedicel, while only *VviHSP23.6* was up-regulated at this stage in pulp and skin while *VviHSP20* was down-regulated in these tissues. In citrus, the concomitant up-regulation of AQPs and HSPs was related to the reduction of oxidative stress risks under drought [64]. These results suggest that canopy architecture manipulation (MSP) activated, rather than repressed, physiological processes by enhancing the onset of oxidative stress in PS berries at the pulp and skin level. This assumption is corroborated by the down-regulation of *VviHSP20* at this stage, which has been previously shown to be repressed by H_2_O_2_ in Kyhoto berries [65].

*VviWRKY40* acts as a transcriptional repressor that, by binding to the *GT14* promoter, represses its activity and impairs the biosynthesis of monoterpenoids [66,67]. *VviWRKY40* expression was also shown to be down-regulated by ABA [67]. Its up-regulation under MSP treatment at VER in the pulp and skin corroborates the hypothesis of a metabolic delay of the onset of maturation in MSP berries. Interestingly, in pulp, *VviWRKY40* is down-regulated in FM_east_, while it is up-regulated in FM_west._ This points towards a delay of terpenoid accumulation in shaded berries on one side. In addition, it also suggests that, in the same plant, MSP applied on the west side of the canopy also modulates the microclimate of berries located on the east side. In fact, vertically upward shoots of VSP on the west side act as barriers to direct solar radiation after solar noon to berries located on the east side of the canopy. However, when leaning the shoots (MSP), this barrier is reduced, and berries located on the east side become more prone to direct solar radiation in the afternoon. Overall, the MSP agronomic practice may lead to an increase in the expression of *VviGT14* on the east side and a reduction of expression on the east side and, consequently, to berries with different amounts of glycosylated monoterpenoids.

## 4. Materials and Methods

### 4.1. Field Trial and Plant Material

The experimental research was conducted from June to September 2019 and 2020, in a vineyard located at Tapada da Ajuda, Lisbon (38°42′27.5″ N, 9°10′56.3″ W and 62 m above sea level). The vineyard has an area of 1.7 ha and belongs to the Instituto Superior de Agronomia (ISA). It was planted in 2006 with a north–south row orientation and a density of 4000 plants/ha, with 1.0 m between plants and 2.5 m between rows. The training system is vertical shoot positioning (VSP) spur pruned in a unilateral Royat cordon, with the plants uniformly pruned to 12–14 nodes per vine, and the rootstock is 1103 Paulsen. The soil is a clay loam, with 1.6% organic matter and a pH of 7.8 [68], and the climate is mesothermic [69]. A meteorological station installed in the vineyard where the study was conducted enabled monitoring of the monthly mean precipitation values and the mean, maximum, and minimum temperatures in the two seasons of the trial (accessed through www.meteoagri.com, last accessed on 21 September 2020, and shown in Figure S2). Soil water probes placed in the vineyard enabled the continuous monitoring of the soil water content during the season (https://www.aquacheckweb.com/login.html, last accessed on 29 August 2020). The vineyard was drip-irrigated, with drip irrigation lines in the center of the row and consisting of pressure-compensating 2.5 L h^−1^ emitters at 1.0 m spacing (one per vine positioned between two adjacent vines). Irrigation began in June (BBCH 69, end of flowering), and water was kept at readily available levels until VER to allow for early berry growth without water stress. After this, irrigation was managed to obtain values of predawn leaf water potential of ≈−0.3 MPa, a mild stress that promotes flavor accumulation, thus enhancing berry quality. Irrigation stopped a week before harvest, at BBCH 89. The inter-row was managed with resident vegetation that was mowed in the spring and left on the ground as mulch.

The experiment was conducted on eight rows of the grapevine (*Vitis vinifera* L.) variety Muscat of Alexandria (syn. Moscatel Graúdo). The experimental design was a randomized complete block with two treatments and four replicates per treatment. The treatments consisted of conventional VSP training, with two pairs of movable wires as a control, and the shaded treatment (modulate shoot positioning, MSP), an adaptation of the conventional VSP, was conducted by withdrawing the upper movable wire at the west side of the canopy to promote the downwards re-positioning of shoots to provide shading of the western-facing bunches. It was decided not to curve the shoots to both sides to prevent the opening of the canopy and, therefore, the exposure of the central bunches. The shoots were not trimmed. In each replicate, six plants were selected for analysis, and the beginning of the MSP treatment took place at the berry touch stage (BBCH 79), which occurred in the middle of June in both seasons. Clusters were monitored and tissues were sampled at pea size (PS), veraison (VER), and full maturation (FM).

### 4.2. Histology

In both seasons, the peduncle samples were taken from four clusters at three different times according to the phenological stage of the vines (PS, VER, and FM). In each sample, a 0.5–1 cm long segment of the upper-middle zone of the stalk of the cluster (peduncle) of the same clusters where the hydraulic measurements were made, i.e., 4 segments from the control treatment (VSP) and 4 segments from the shading treatment (MSP). All collected samples were immediately fixed in FAE (formalin–acetic acid–ethanol, in a 2:1:10 proportion) [70]. Afterward, they were dehydrated in ascending ethanol solutions, clarified with tertiary butyl alcohol, and embedded in paraffin plasticized + DMSO pellets (M.P. 56–58 °C, Panreac-AppliedChem, ITW Reagents, Chicago, IL, USA) by the paraffine/TBA method [70]. Transverse sections of 10 μm thick were cut using a rotary microtome (Leica RM2255).

Two staining procedures allowed us to analyze the cluster peduncle anatomy; after deparaffinization with Cytrosol (Panreac-AppliedChem) and dehydration with an ethanol-decreasing series, the sections were stained using either (i) single staining with a 0.05% (*w*/*v*) aqueous solution of Toluidine blue (Sigma-Aldrich) or with a 0.05% (*w*/*v*) aqueous solution of Safranine O DC (Sigma-Aldrich, St. Louis, MO, USA) or (ii) double staining with a 1% aqueous solution of Safranin for 1 min, washed with distilled water, and then stained with a 0.5% (*w*/*v*) aqueous solution of Astra blue (Sigma-Aldrich) for 20 min and washed with distilled water [71]. After that, sections were dehydrated with increasing ethanol concentrations and mounted in a Eukitt mounting medium (Sigma-Aldrich). Finally, all sections were observed and photographed using a Nikon SMZ1000 stereomicroscope (0.8×) and a Nikon Eclipse 50i light microscope (at different magnifications), both coupled with a Nikon DS-Fi1 camera and analyzed with NIS-Elements Advanced Research v. 3.22.15 Software (Nikon Instruments Inc., Tokyo, Japan).

For each treatment and sampling stage, the total section area and the areas occupied by the different tissues (cortex, vascular cylinder, xylem, phloem, and pith) were measured on microphotographs of peduncle cross sections using Image J software v. 2.14.0/1.54f (as exemplified in Figure S3). These morphometric measurements were performed on five complete cross sections of four peduncles per treatment and sampled with a minimum spacing of at least 150 µm. Similarly, the quantitative characteristics of the primary and secondary xylem vessels (area, perimeter, and diameter) were determined on a 90° circular sector of the same sections [72].

### 4.3. Hydraulic Conductance

Considering the grapevine berries’ asynchronous growth and ripening within the same cluster, especially at the VER stage [73], and to avoid biased results in hydraulic conductance, three extra sampling points (in addition to PS and FM) were made for these measurements: the beginning of veraison (VER_i_), the end of the veraison (VER_f_), and mid-ripening (MR). Visually healthy clusters on the west side of each replicate of both treatments (*n* = 4 plants) were collected in the early morning (to ensure minimal xylem tension). The clusters were excised underwater to avoid artificially induced bias in hydraulic conductance (K_h_). The samples were then bagged and transported in water to the lab at 4 °C for hydraulic analyses. For each cluster, berries were counted and excised (without pedicel) underwater. The rachis length was assessed with image analysis software (ImageJ). The upper cross-section diameter of the cluster, cluster fresh weight (FW), and dry weight (DW), as well as the pedicel number, FW, and DW, were also assessed.

Cluster peduncles were wrapped in Teflon tape and connected to the flow meter by their proximal ends, and K_h_ was measured at low pressure (2^–4^ × 10^−3^ MPa) when the flow reached a stable value. A perfusion solution of ultra-pure, deionized, degassed, and filtered (0.2 µm) water was used in K_h_ measurements.

Hydraulic conductance (K_h_, kg s^−1^ MPa^−1^), calculated as the ratio between the flow through each cluster and the corresponding hydrostatic pressure gradient, was measured in the whole cluster (rachis+pedicels), following Sperry et al. [74], with a high precision flow meter, XYL’EM (Embolism Meter, Bronkhorst, Montigny-Les-Cormeilles, France). Hydraulic conductance was normalized to the length of each cluster (hydraulic conductivity, kg s^−1^ m MPa^−1^). The hydraulic conductivity was converted to cluster-specific hydraulic conductivity (K_rachis+pedicels_) by dividing by the cluster sectional area (m^2^) and by the berry number. To assess the pedicel resistance, a second Kh measurement was conducted after excising all pedicels under water. The hydraulic conductivity was converted to cluster-specific hydraulic conductivity (K_rachis_) by dividing by the cluster sectional area (m^2^) and by the pedicel number.

It is important to note that during 2019, hydraulic measurements were only performed on rachis+pedicels at PS and VER_i_, while measurements of the rachis without pedicels were only performed at VER and FM. In 2020, hydraulic measurements were performed both in rachis+pedicels and in rachis at all phenological stages.

### 4.4. RNA Extraction

Cluster fractions were collected in grapevines of both treatments, on the west side of the canopies in PS, VER, and FM. Also, in FM, cluster fractions were collected both at the east and the west sides of the canopy. Samples were kept at −80 °C until processing. Samples from pedicels, pulp, and skin (except in PS where the whole berry was used) were ground in the presence of liquid nitrogen with a mortar and pestle. Total RNA was extracted using the Spectrum™ Plant Total RNA kit (Sigma-Aldrich, St. Louis, MO, USA). Nucleic acid concentration was quantified spectrophotometrically using a Take3 plate and the software Gen5 v1.09 in a Synergy HT (Bio-Tek Instruments, Winooski, VT, USA). The quality of the RNA extracted was evaluated through the ratios A_260_/A_280_ and A_260_/A_230,_ and the RNA integrity was assessed through 1.5% agarose-gel electrophoresis under denaturing conditions.

### 4.5. cDNA Synthesis for qPCR

RNA samples were treated with RQ1 RNase-Free DNase (Promega, Madison, WI). cDNA was synthesized from 1 µg of total RNA using oligo(dT)_20_ in a 20 µL reaction volume using RevertAid Reverse Transcriptase (Thermo Fisher Scientific, Waltham, MA, USA) according to the manufacturer’s recommendations. cDNA was tested for gDNA contamination in PCRs using intron spanning primers that yield a 229 bp amplicon in cDNA and a 547 amplicon in gDNA. Amplicon sizes were compared in 2% agarose gels together with the molecular weight marker 1Kb^+^ (Thermo Fisher Scientific), and no gDNA contamination was detected. cDNA was stored at −20 °C until further use.

### 4.6. qPCR

Primers for AQP, HSPs, and other stress-responsive genes were obtained from the literature or from previous studies. Primers were designed with the software Beacon Designer v2.0 (Premier Biosoft, San Francisco, CA, USA) using a primer length of 20 ± 2 bp, a melting temperature of 60 ± 2 °C, a guanine–cytosine content of circa 50%, and an expected amplicon size of 180–280 bp. See Table S2 for sequences, amplicon size, and the respective references. Real-time qPCR reactions were performed in 96-well clear plates (Bio-Rad, Hercules, CA, USA), using an IQ5 Real Time PCR (Bio-Rad, Hercules, CA, USA) with five biological replicates. The 20 µL reaction mixture was composed of 1 µL cDNA diluted 50 fold, 0.5 µM of each gene-specific primer, and 10 µL master mix (SsoFast_EvaGreen Supermix, Bio-Rad, Hercules, CA, USA). Amplification of PCR products was monitored via intercalation of the Eva-Green present in the master mix. The following program was applied: initial polymerase activation, 95 °C, 3 min, then 40 cycles at 94 °C for 10 s (denaturation), 60 °C for 20 s (annealing), and 72 °C for 15 s (extension), followed by a melting curve analysis to confirm the accurate amplification of target gene fragments and the absence of primer dimers. The PCR products were run on 2% agarose gels to verify that there was only one amplicon of the expected size. PCRs with each primer pair were also performed on samples lacking a cDNA template in triplicate (no template controls). To assess the amplification efficiency of the candidate genes, identical volumes of the cDNA samples were diluted and used to generate five-point standard curves based on a five-fold dilution series (1; 1:5; 1:25; 1:125; and 1:625) in triplicate. Amplification efficiency (E) is calculated as E= 10(−1/a) − 1, with “a” being the slope of the linear regression curve (y = a log(x) + b) fitted over the log-transformed data of the input cDNA dilution (y) plotted against the respective quantification cycle (Cq) values (x). E-values of the target genes were considered comparable when they did not exceed 100 ± 10%, corresponding to a standard curve slope of 3.3 ± 0.33. All cDNA samples were diluted 50 fold and were amplified in duplicate in two independent PCR runs.

To generate a baseline-subtracted plot of the logarithmic increase in the fluorescence signal (ΔRn) versus the cycle number, baseline data were collected between cycles 5 and 17. All amplification plots were analyzed with an Rn threshold of 0.2 at the beginning of the region of exponential amplification to obtain Cq (quantification cycle), and the data obtained were exported into an MS Excel workbook (Microsoft Inc., Albuquerque, New Mexico, USA) for analysis. The reference genes used were *ACT2*, *TIF*, *TIF-GTP* [75], and *ACT1.*

### 4.7. Statistical Analysis

The statistical analysis of the morphometric measurements of the microphotographs and hydraulic conductivity was performed using the statistical package IBM SPSS Statistics v. 22. To study the effect of treatment in the same sampling, Student’s *t*-test (*p* < 0.05) was performed, and to analyze the effect of maturation in each year, an ANOVA test was performed, followed by Tukey’s HSD multiple comparisons test (*p* < 0.05).

For the relationship between the expressions of the selected genes and the reference genes, the relative quantity values were transformed into log_2_ and tested through an ANOVA in software R v4.2.3 [76]. When the *p*-value of the ANOVA was lower than 0.05, a Tukey test was performed, and statistically significant differences were accepted for a *p*-value lower than 0.05.

GraphPad Prism 10 for Windows (GraphPad Software, San Diego, CA, USA) was used for figure creation.

## 5. Conclusions

This study highlights the role of shading, through canopy architecture manipulation, in modulating berry–water relations and influencing the response to stress during fruit development. This role is also influenced by environmental conditions, likely becoming more pronounced in seasons with more stressful events, such as drought, precipitation distribution anomalies, high average temperatures, and/or heat waves.

Grape-cluster hydraulics is very dynamic in the variety Muscat of Alexandria, changing according to the phenological stages while reflecting each year’s conditions for berry growth. Veraison is shown to be a pivotal phenological stage for hydraulic adjustment in grapevine clusters, with a reduction in xylem diameter and an increase in hydraulic resistance, confirming the shift in water transport into the berry from the xylem to the phloem. The differential expression of several genes coding AQPs confirms this shift in water transport and in regulating xylem hydraulic resistance. In fact, canopy-architecture manipulation (MSP) modulates the expression of the different AQP genes in all tissues. Water transport, mediated by AQP, was down-regulated in the pedicels at full maturation, while in the pulp it was enhanced, especially at veraison and in the shaded treatment. Conversely, the shaded treatment had little influence on AQP expression in berry skins. The stress response was heightened in all berry tissues during the whole season, with an enhancement of the onset of oxidative stress in pea-size berries in the shaded treatment.

Overall, this study highlights that a simple agronomical management, such as canopy manipulation, induces changes in water relations within the berry cluster during berry maturation in the same plant. Shading affects the ripening process, with a likely consequence on sugar, amino acid, and phenolic compound accumulation.

## Data Availability

The original contributions presented in the study are included in the article/Supplementary Materials; further inquiries can be directed to the corresponding author/s.

## Supplementary Materials

The following supporting information can be downloaded at https://www.mdpi.com/article/10.3390/plants13111500/s1, Figure S1: Stomata and Lenticels; Figure S2: Monthly temperatures and precipitation during the years of 2019 and 2020; Figure S3: Morphometric peduncle measurements and image analysis; Table S1: Morpho-anatomical parameters and quantitative characteristics of vascular tissue of Muscat of Alexandria peduncle; Table S2: List of Primers utilized in RT-qPCR.
