# Supplementary material for: Canopy Architecture and Sun Exposure Influence Berry Cluster–Water Relations in the Grapevine Variety Muscat of Alexandria
Source: Plants (Basel). 2024 May 29;13(11):1500. doi: 10.3390/plants13111500 (PMC11174960; doi:10.3390/plants13111500)
Supplement: Supplementary file 1 [file plants-13-01500-s001.zip › SupplementalFigures.pdf]

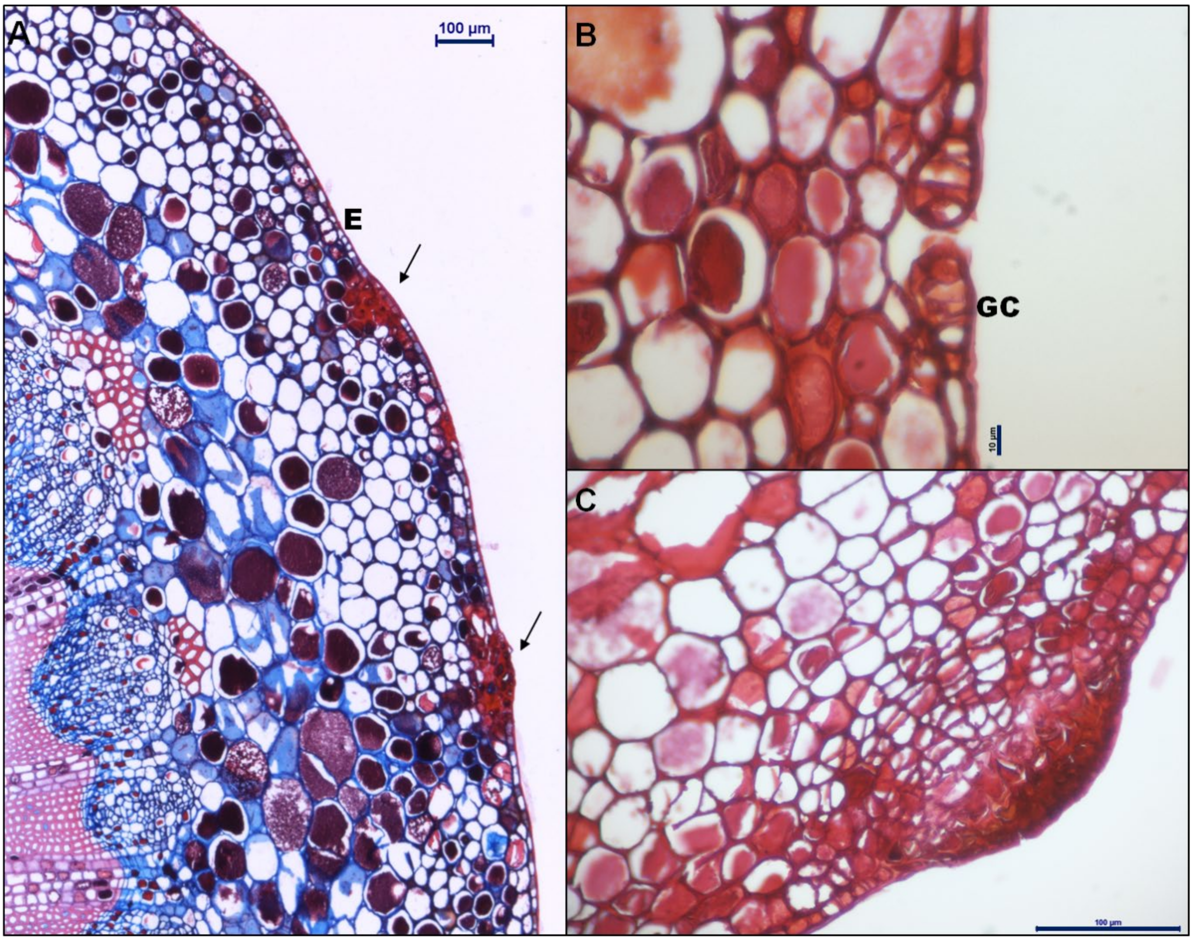

Figure S1: Stomata and Lenticels. A) Cross section stained with Safranin and Astra Blue. Lignified cells turn reddish with safranin. Epidermis is interrupted by the formation of stomata and lenticels. Scale bar: 100 µm. B) Details of stomata stained with Safranin. Scale bar: 10 µm. C) Details of lenticel stained with Safranin. Scale bar: 100 µm. Arrows indicate lenticels. Abbreviations: E: epidermis; GC: stomata guard cell.

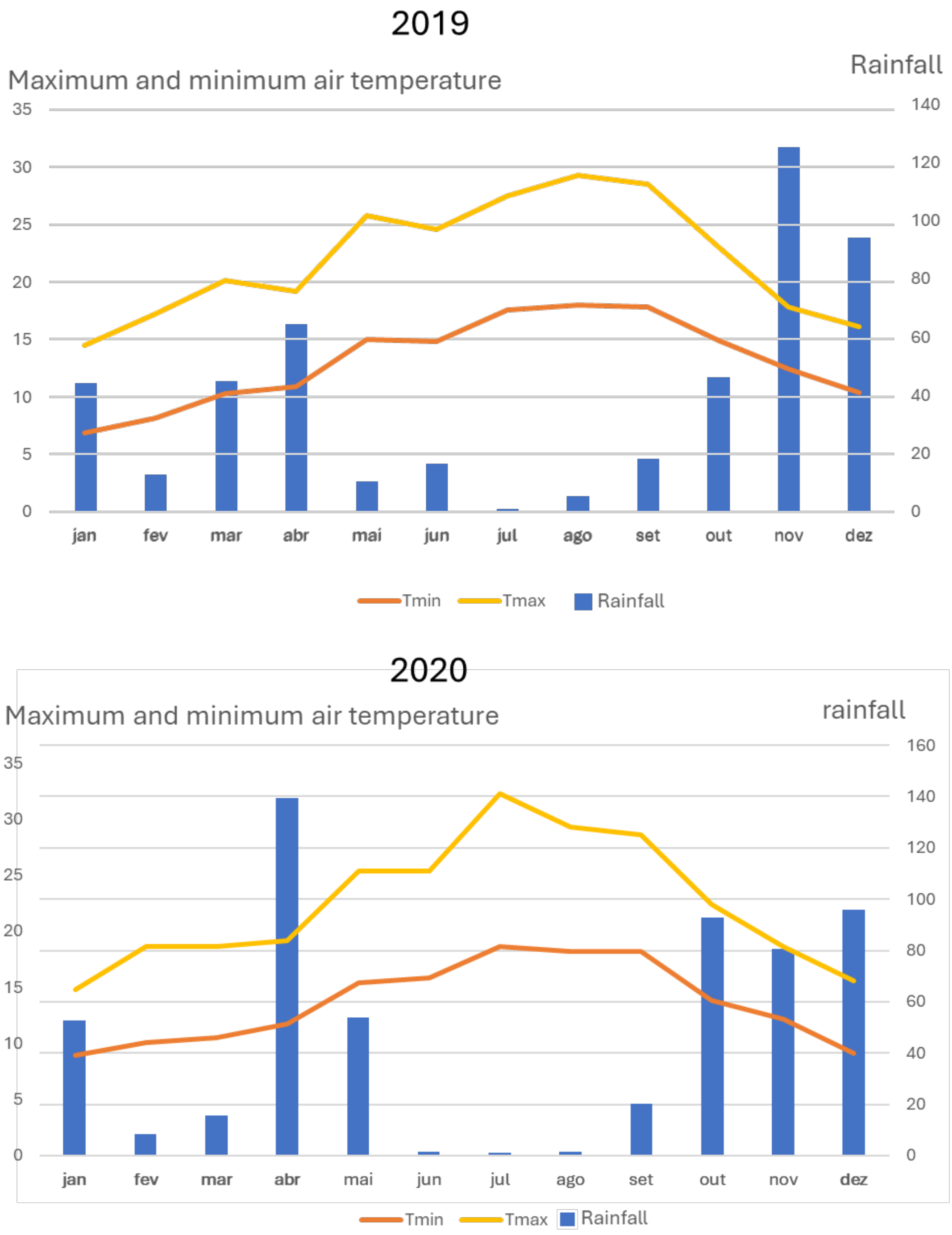

Figure S2: Monthly maximum (Tmax) and minimum (Tmin) temperatures and total monthly precipitation during the years of 2019 and 2020.

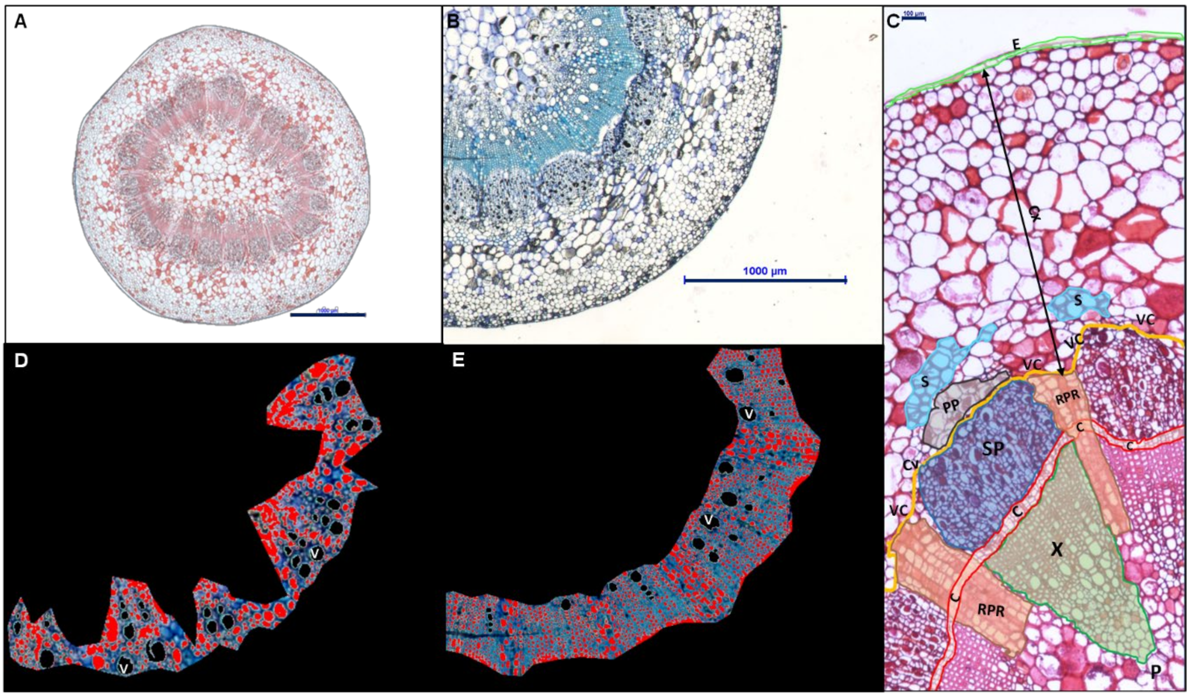

Figure S3: Morphometric measurements and image analysis. A) Measurement of complete peduncle section area stained with Safranin. Scale bar: 1000 µm. B) A 90° circular sector of the section stained with Toluidine Blue. Scale bar: 1000 µm C) Delimited areas used during *Image J* measurement process. Scale bar: 100 µm. D) and E) Final aspect of microphotographs treated with an *ImageJ* thresholding method for primary and secondary xylem vessels quantification. Selected vessels for measurements are filled of black.

Abbreviations (of colored areas and lines): C: cambium; Cx: cortex; E: epidermis; P: pith; PP: primary phloem; RPR: radiomedullary parenchyma rays; S: sclerenchyma; SP: secondary phloem; X: xylem; v: vessel; VB: vascular bundle; VC: vascular cylinder area delimited line.
